# Using PROMIS for measuring recovery after abdominal surgery: a pilot study

**DOI:** 10.1186/s12913-018-2929-9

**Published:** 2018-02-20

**Authors:** Eva van der Meij, Johannes R. Anema, Judith A. F. Huirne, Caroline B. Terwee

**Affiliations:** 1 Department of Obstetrics and Gynecology, VU University Medical Center, van der Boechorststraat 7, 1081 BT Amsterdam, The Netherlands; 2 Department of Public and Occupational Health, Amsterdam Public Health research institute, VU University Medical Center, van der Boechorsstraat 7, 1081 BT Amsterdam, The Netherlands; 3 Department of Epidemiology and Biostatistics, Amsterdam Public Health research institute, VU University Medical Center, Amsterdam, The Netherlands

**Keywords:** PROMIS, Postoperative recovery, Abdominal surgery, Inguinal hernia surgery, Cholecystectomy, Hysterectomy, Adnexal surgery

## Abstract

**Background:**

To assess the construct validity and responsiveness of the PROMIS Physical Function v1.2 short form 8b (PROMIS-PF), and the PROMIS Ability to Participate in Social Roles and Activities v2.0 short form 8a (PROMIS-APS) in postoperative recovery.

**Methods:**

An observational pilot study was conducted in which 30 patients participated, undergoing various forms of abdominal surgery. Patients completed the PROMIS-PF and PROMIS-APS, the Short Form 36 Health Survey (SF-36) and the World Health Organization Disability Assessment Schedule 2.0 (WHODAS) at several time points before and after surgery. The construct validity and responsiveness of the two PROMIS short forms were evaluated by testing pre-defined hypotheses and were considered adequate when at least 75% of the data was consistent with the hypotheses. Construct validity was evaluated by calculating Spearman correlations and the responsiveness by calculating effect sizes.

**Results:**

6/7 (85.7%) of the results were consistent with the hypotheses supporting the construct validity of the PROMIS-PF. For the PROMIS-APS this was the case in 7/15 (46.7%) of the results. For the PROMIS-PF, 6/7 (85.7%) of the results were consistent with the hypotheses, supporting responsiveness. Regarding the responsiveness of the PROMIS-APS, only 7 out of 13 (53.8%) of these results were consistent with the hypotheses.

**Conclusions:**

This study supported the construct validity and the responsiveness of the PROMIS-PF v1.2 short form 8b for measuring recovery in abdominal surgery. Considering the major advantages of PROMIS, we recommend the use of the PROMIS-PF in abdominal surgery.

## Background

Measuring recovery after surgery has become increasingly important over the past years. This is due to the fact that new surgical techniques have been developed and interventions to speed up recovery are increasingly popular, which means that the number of comparative studies in postoperative care is growing [1–4]. However, postoperative recovery is a complex and multi-dimensional construct and the recovery process varies among patients [5, 6]. A variety of instruments is currently being used to measure recovery after surgery, capturing different aspects of the recovery process, such as physical function, pain, or participation in society [7–9]. These questionnaires are often quite long and time consuming to complete, which is experienced as burdensome by patients [10, 11]. Furthermore, all patients need to complete the same questions, while not all questions are relevant to all patients. It is not clear either whether these instruments are sensitive enough to measure relevant changes in post-operative function from a patient’s perspective [10, 12, 13].

A promising alternative is the Patient-Reported Outcomes Measurement Information System (PROMIS) [14]. The PROMIS initiative has developed a new innovative generic assessment system for measuring patient-reported health, consisting of Item Response Theory (IRT)-based item banks, which are large sets of questions (items) that all measure the same construct, such as physical function or participation. An advantage of using item banks is that relevant items for a specific patient (group) can be selected from an item bank and administered as short forms, consisting of a fixed set of 4–10 items. This has the potential to personalize questionnaires by selecting only those items which are relevant for a specific patient or population. This is particularly interesting for measuring postoperative recovery, since the postoperative recovery process is different for each individual.

An ultimate form of personalization is to administer item banks through Computerized Adaptive Testing (CAT). With CAT items are selected from an item bank based upon the respondent’s answers to previous questions. A main advantage of CAT is that patients get more relevant questions and fewer questions are required to get a reliable score. However, a computer and specific CAT software are required.

The PROMIS instruments have been validated in several populations and countries and shown to have good measurement properties [15]. Also, PROMIS has shown to be responsive to changes in surgical patients [16–18]. However, construct validity and responsiveness have not been assessed in patients undergoing abdominal surgery and no longitudinal validation study has yet been performed in the Netherlands. Since CAT software was not yet available at the start of this study, we aimed to evaluate the construct validity and responsiveness of the PROMIS Physical Function v1.2 short form 8b (PROMIS-PF) and the PROMIS Ability to Participate in Social Roles and Activities v2.0 short form 8a (PROMIS-APS) in patients undergoing commonly applied minor surgical abdominal procedures.

## Methods

### Study design

Data was used from an observational pilot study in 30 patients undergoing minor surgical abdominal procedures. The original aim of the pilot study was to evaluate the feasibility of using an accelerometer in the postoperative course after abdominal surgery, in preparation for a clinical trial [19, 20]. A sample size of 30 was considered appropriate for the feasibility testing of the accelerometer. Questionnaires were completed by all patients in the pilot study and could be used for assessing the construct validity and responsiveness of the PROMIS short forms. The study was approved by the local medical ethics committee under registration number 2014.364 and funded by ZonMw (project number 837002409), an organization for health research and development in the Netherlands. Patients who fulfilled the inclusion criteria and who were willing to participate, signed informed consent.

### Participants

Patients were recruited from the surgical waiting lists of two participating teaching hospitals in Amsterdam, the Netherlands between September 2014 and July 2015. Patients undergoing one of the following types of surgical procedures were eligible for the study: laparoscopic hysterectomy, laparoscopic adnexal surgery, laparoscopic cholecystectomy and laparoscopic inguinal hernia repair. Laparoscopic adnexal surgery, laparoscopic cholecystectomy and laparoscopic inguinal hernia repair were classified as minor surgical procedures and laparoscopic hysterectomy as an intermediate surgical procedure. This subdivision is commonly used in gynecologic surgery [7, 21]. Exclusion criteria were: (suspicion of) malignancy, deep infiltrating endometriosis, a waiting period of less than one week for surgery, lack of understanding of the study information, insufficient Dutch language proficiency, or lack of informed consent.

### Measurements

Participants were asked to complete four questionnaires electronically at four different moments (during the month before surgery (T0), one week after surgery (T1), three weeks after surgery (T2) and five weeks after surgery (T3)) (Table 1).

**Table 1:** Assessment of outcome measures

	One week before surgery T0	One week after surgery T1	Three weeks after surgery T2	Five weeks after surgery T3
PROMIS -PF	X	X	x	x
PROMIS-APS	X	X	x	x
WHODAS	X	X	x	x
SF-36	X		x	x

*PROMIS-PF* PROMIS - Physical Function v1.2 short form 8b, *PROMIS-APS* PROMIS Ability to Participate in Social Roles and Activities v2.0 short form 8a, *WHODAS* WHO Disability Assessment Schedule 2.0, *SF-36* The Short Form (36) Health Survey

#### PROMIS - physical function v1.2 short form 8b (PROMIS-PF)

The PROMIS-PF item bank consists of 121 items and measures self-reported capability rather than actual performance of physical activities. This includes the functioning of one’s upper extremities (dexterity), lower extremities (walking or mobility), and central regions (neck, back), as well as instrumental activities of daily living, such as running errands http://www.assessmentcenter.net/documents/PROMIS%20Physical%20Function%20Scoring%20Manual.pdf. The PROMIS short form v1.2 8b was derived from the PROMIS-PF function item bank, and contains eight questions assessing limitations in daily physical activities. This IRT-based item bank has been developed and validated in the US and translated into Dutch-Flemish [22, 23]. Validation studies performed in Dutch patients confirmed the unidimensionality and underlying calibration of the IRT model [24–26]. Scores are expressed as T-scores, representing a standardized score with a mean of 50 (corresponding to the mean score in the US general population) and a standard deviation (SD) of 10. Higher scores mean better physical function.

#### PROMIS ability to participate in social roles and activities v2.0 short form 8a (PROMIS-APS)

The PROMIS-APS item bank contains 35 items and assesses the perceived ability to perform one’s usual social roles and activities http://www.assessmentcenter.net/documents/PROMIS%20Physical%20Function%20Scoring%20Manual.pdf. The short form V2.0 8a consists of eight questions and was derived from the PROMIS-APS item bank. This IRT-based item bank has been developed and validated in the US and translated into Dutch-Flemish [27, 28]. Validation studies performed in Dutch patients undergoing rehabilitation and in the Dutch general population confirmed the unidimensionality and underlying calibration of the IRT model (personal communication, manuscripts in preparation). Scores are expressed as T-scores, representing a standardized score with a mean of 50 (corresponding to the mean score in the US general population) and a standard deviation (SD) of 10. Higher scores mean better ability to participate.

#### WHO disability assessment schedule 2.0 (WHODAS)

The WHODAS is a self-report questionnaire developed by the World Health Organization (WHO), containing 36 questions divided into six subscales [29]:

- Cognition – understanding & communicating (WHO-CG, 6 items)
- Mobility– moving & getting around (WHO-MO, 5 items)
- Self-Care– hygiene, dressing, eating & staying alone (WHO-SC, 4 items)
- Getting Along– interacting with other people (WHO-GA, 5 items)
- Life Activities– domestic responsibilities, leisure, work & school (WHO-LA, 4 items), divided in the Household subscale (WHO-LA-H) and the Work subscale (WHO-LA-W, 4 items)
- Participation– joining in community activities (WHO-PART, 8 items)

Construct validity and responsiveness of the WHODAS was supported in people with different health conditions across different cultures http://apps.who.int/iris/bitstream/10665/43974/1/9789241547598_eng.pdf [30]. Higher scores indicate more impairment in the constructs being measured.

#### The short form (36) health survey (SF-36)

The SF36 contains 36 questions measuring eight constructs:

- Physical Functioning (SF-PF, 10 items)
- Emotional Role Functioning (SF-ERF, 3 items)
- Physical Role Functioning (SF-PRF, 4 items)
- Bodily Pain (SF-BP, 2 items)
- Mental Health (SF-MH, 5 items)
- Vitality (SF-VT, 4 items)
- Social Functioning (SF-SF, 2 items)
- General Health (SF-GH, 5 items)

The Dutch version of the questionnaire was used, which was validated in a Dutch general population. Multitrait scaling analysis confirmed the hypothesized scale structure of the SF-36 and internal consistency was high. Known-group comparisons yielded consistent support for the validity of the SF-36 [23]. Higher scores represent more of the construct being measured.

### Statistical analyses

SPSS version 20.0 was used to analyze the data. Baseline characteristics were presented using descriptive statistics. To evaluate construct validity and responsiveness of the PROMIS-PF and the PROMIS-APS, predefined hypotheses (by EM and CT) were tested:

#### Hypotheses regarding the construct validity of the PROMIS-PF

1, 2, 3, 4: The PROMIS-PF has a high correlation (> 0.7) with the Mobility subscale of the WHODAS (**WHO-MO**) on each time point (T0, T1, T2, T3).

5, 6,7: The PROMIS-PF has a high correlation (> 0.7) with the Physical Functioning subscale of the SF-36 (**SF-PF**) on each time point (T0, T2, T3).

#### Hypotheses regarding the construct validity of the PROMIS-APS

1, 2, 3, 4: The PROMIS-APS has a high correlation (> 0.7) with the Life Activities-Household subscale of the WHODAS (**WHO-LA-H**) on each time point (T0, T1, T2, T3).

5, 6, 7, 8: The PROMIS-APS has a high correlation (> 0.7) with the Life Activities-Work subscale of the WHODAS (**WHO-LA-W**) on each time point (T0, T1, T2, T3).

9, 10, 11, 12: The PROMIS-APS has a high correlation (> 0.7) with the Participation subscale of the WHODAS (**WHO-PART**) on each time point (T0, T1, T2, T3).

13, 14, 15: The PROMIS-APS has a high correlation (> 0.7) with the Physical Role Functioning subscale of the SF-36 (**SF-PRF**) on each time point (T0, T2, T3).

#### Hypotheses regarding the responsiveness of the PROMIS-PF

1, 2, 3: Intermediate surgical procedures show larger change in physical function scores between the consecutive time points (T0-T1, T1-T2, T2-T3) than minor surgical procedures.

4, 5, 6: The PROMIS-PF is equally or more responsive (at most 0.05 smaller effect size) than the **WHO-MO** subscale of the WHODAS between the consecutive time points (T0-T1, T1-T2, T2-T3)7: The PROMIS-PF is equally or more responsive (at most 0.05 smaller effect size) than the **SF-PF** subscale of the SF-36 between the consecutive time points (T2-T3)

#### Hypotheses regarding the responsiveness of the PROMIS-APS

1, 2, 3: Intermediate surgical procedures show larger change in participation scores between the consecutive time points (T0-T1, T1-T2, T2-T3) than minor surgical procedures.

4, 5, 6: The PROMIS-APS is equally or more responsive (at most 0.05 smaller effect size) than the **WHO-LA-H** of the WHODAS between the consecutive time points (T0-T1, T1-T2, T2-T3)

7, 8, 9: The PROMIS-APS is equally or more responsive (at most 0.05 smaller effect size) than the **WHO-LA-W** of the WHODAS between the consecutive time points (T0-T1, T1-T2, T2-T3)

10, 11, 12: The PROMIS-APS is equally or more responsive (at most 0.05 smaller effect size) than the **WHO-PART** of the WHODAS between the consecutive time points (T0-T1, T1-T2, T2-T3)

13: The PROMIS-APS is equally or more responsive (at most 0.05 smaller effect size) than the **SF-PRF** subscale of the SF-36 between the consecutive time points (T2-T3)

Spearman correlations were calculated for assessing construct validity. Construct validity was considered sufficient when at least 75% of the results were consistent with the hypotheses. Responsiveness was evaluated by comparing the effect sizes between the PROMIS short forms and the subscales of the WHODAS and SF-36. Effect sizes were calculated by dividing the change score between two consecutive time points by the standard deviation (SD) of the first time point. Responsiveness was considered sufficient when at least 75% of the results were consistent with the hypotheses.

## Results

### Participants

Thirty patients (34.9%) gave consent to participate. No statistically significant differences regarding age, gender, social economic status and type of surgery were found between patients who participated and those who did not. More details about the inclusion process are described in our related article [31]. All questionnaires were completed without missing values on each time point by all participants, except for the last questionnaire (T3), which one participant failed to complete. Baseline characteristics of the participants are presented in Table 2. Six participants underwent adnexal surgery, four patients inguinal hernia repair, three patients a cholecystectomy and twelve a hysterectomy. Most patients were female (76.7%) and the mean age was 45.3 years. Mean PROMIS scores during the month before surgery (T0) were close to 50, comparable to the average general population (Table 2).

**Table 2:** Baseline characteristics of the patients

Gender (*n* %)	
- Male	7 (23.3%)
- Female	23 (76.7%)
Age (mean sd)	45.3 (8.8)
Level of education (*n* %)^a^	
- Low	6 (20.0%)
- Medium	12 (40.0%)
- High	12 (40.0%)
Employment status (*n* %)	
- Employed	25 (83.3%)
- Unemployed	5 (16.7%)
Type of surgery (all laparoscopic) (*n* %)	
- Minor surgical procedures	17 (56.7%)
- Adnexal surgery	7
- Inguinal hernia repair	5
- Cholecystectomy	5
- Intermediate (hysterectomy)	13 (43.3%)
ASA classification (mean sd)	*n = 24*
1	16 (66.7%)
2	7 (29.2%)
3	1 (4.2%)
BMI (mean sd)	25.9 (4.6)
Mean PROMIS-PF T-score (SD) before surgery (T0)	49.4 (9.5)
Mean PROMIS-APS T-score (SD) before surgery (T0)	50.7 (10.1)

^a^Low = preschool, primary school, lower vocational education Intermediate = secondary education, intermediate vocational education High = higher vocational education, university, postgraduate

*ASA* American Society of Anesthesiologists classification, *BMI* Body Mass Index, *PROMIS-PF* Physical Function short form, *PROMIS-APS* PROMIS Ability to Participate in Social Roles and Activities short form, *SD* Standard Deviation

### Construct validity

In Table 3 the correlations of the PROMIS-PF and of the PROM-APS with each subscale of the SF-36 and WHODAS per time point are presented. For the PROMIS-PF 6/7 (85.7%) of the results were consistent with the hypotheses and the construct validity of the PROMIS-PF was therefore supported. For the PROMIS-APS 7/15 (46.7%) of the results were consistent with the hypotheses and thus the construct validity was not supported.

**Table 3:** Construct validity of the PROMIS-PF and PROMIS-APS

A: Correlations of the PROMIS-PF with the WHO-MO and SF-PF				
	WHO-MO^a^		SF-PF	
T0	**−0.70**		**0.92**	
T1	−0.66		Not measured	
T2	**−0.84**		**0.92**	
T3	**−0.76**		**0.88**	
B: Correlations of the PROMIS-APS with the WHO-LA-H, WHO-LA-W, WHO-PART and SF-PRF				
	WHO-LA-H*	WHO-LA-W*	WHO-PART*	SF-PRF
T0	**−0.71**	**−0.76**	**−0.89**	**0.72**
T1	−0.61	−0.55	− 0.80	Not measured
T2	−0.62	−0.68	− 0.65	0.58
T3	**−0.91**	**−0.76**	− 0.69	0.69

Correct expected correlations are highlighted in bold

T0: one week before surgery. T1: one week after surgery. T2: three weeks after surgery . T3: five weeks after surgery

WHO-MO: Mobility subscale of the WHODAS. SF-PF: Physical Role Functioning subscale of the SF-36. WHO-LA-W: Life Activities-Work subscale of the WHODAS. WHO-LA-H: Life Activities-Household subscale of the WHODAS. WHO-PART: the Participation subscale of the WHODAS. SF-PRF: Physical Role Functioning subscale of the SF-36

^*^Correlations are negative because higher WHODAS scores indicate more impairment regarding mobility

### Responsiveness

**Fig. 1:**
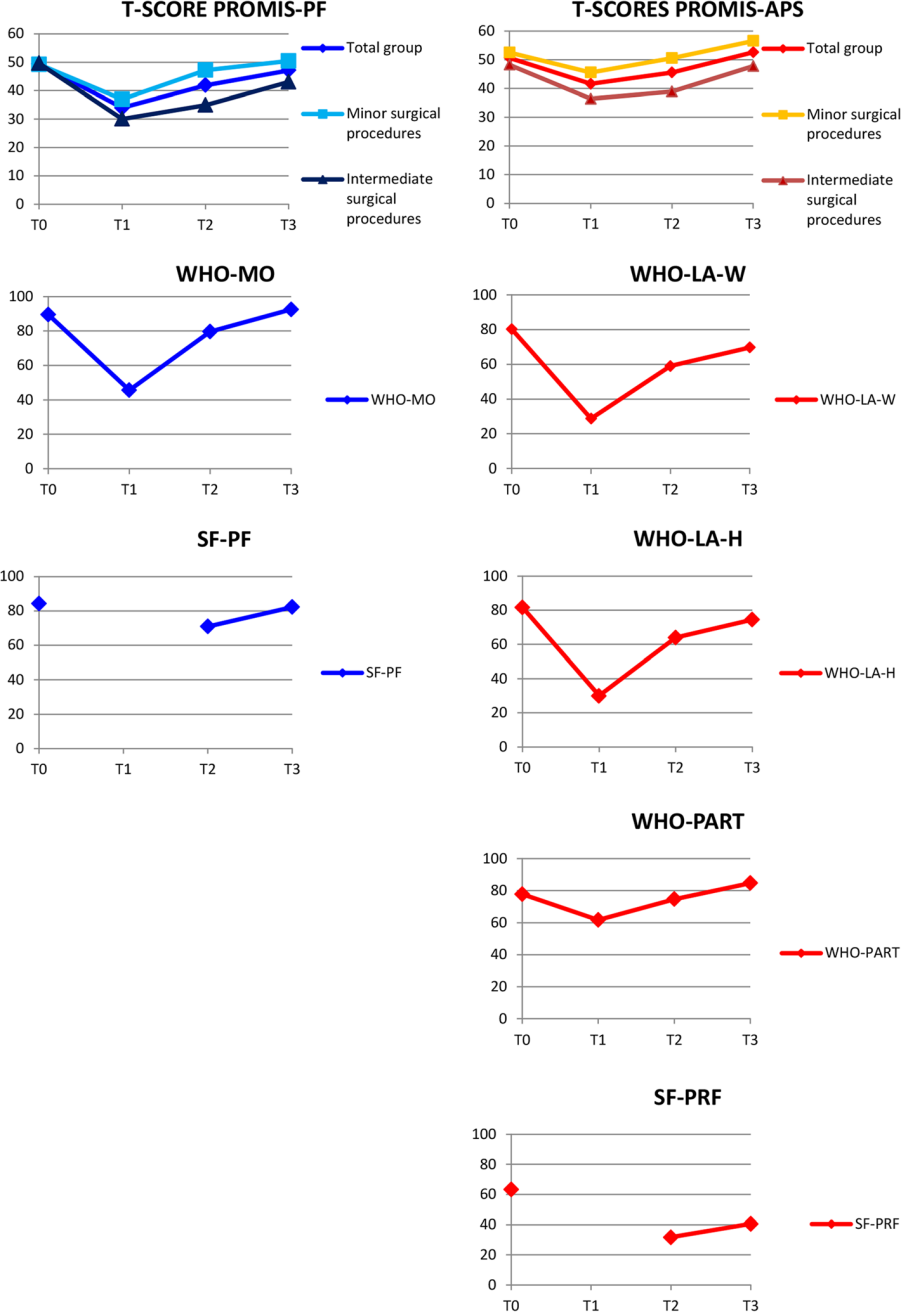
Responsiveness of the PROMIS-PF and the PROMIS-APS. Figure legend: T0: one week before surgery. T1: one week after surgery. T2: three weeks after surgery. T3: five weeks after surgery. PROMIS-PF: Physical Function short form. PROMIS-APS: PROMIS Ability to Participate in Social Roles and Activities short form. WHO-MO: Mobility subscale of the WHODAS. SF-PF: Physical Role Functioning subscale of the SF-36. WHO-LA-W: Life Activities-Work subscale of the WHODAS. WHO-LA-H: Life Activities-Household subscale of the WHODAS. WHO-PART: the Participation subscale of the WHODAS. SF-PRF: Physical Role Functioning subscale of the SF-36

For the PROMIS-PF, six out of seven results (85.7%) were consistent with the hypotheses and thus the responsiveness was supported (Table 4, Fig. 1). Only the WHO-MO was more responsive than the PROMIS-PF between T0 and T1. The responsiveness of the PROMIS-APS was not supported: only 7 out of 13 (53.8%) of the results were consistent with the hypotheses (Table 4, Fig. 1). Remarkable was that the results at the final time period (T2-T3), were all consistent with the hypotheses.

**Table 4:** Responsiveness of the PROMIS-PF and PROMIS-APS

A. PROMIS-PF		
Hypotheses on each time point	Explanation Group or subscale: Effect size Change score (SD)	
Hypothesis: Intermediate surgical procedures show larger change in physical function scores on each time point than minor surgical procedures.		
**T0-T1**	**Intermediate: 2.11** **19.52 (9.25)**	**Minor: 1.23** **12.26 (10.00)**
**T1-T2**	**Intermediate: 1.78** **4.85 (2.73)**	**Minor: 1.27** **10.22 (8.07)**
**T2-T3**	**Intermediate: 1.81** **8.12 (4.48)**	**Minor: 0.26** **2.61 (9.88)**
Hypothesis: The PROMIS-PF is equally or more responsive (at most 0.05 smaller effect size) than the WHO-MO subscale of the WHODAS between the consecutive time points		
T0-T1	PROMIS-PF: 1.62 15.40 (9.52)	Who-MO: 2.49 43.96 (17.62)
**T1-T2**	**PROMIS-PF: 1.11** **7.90 (7.14)**	**Who-MO: 1.16** **33.96 (29.37)**
**T2-T3**	**PROMIS-PF: 0.51** **5.08 (10.01)**	**Who-MO: 0.56** **12.50 (22.44)**
The PROMIS-PF is equally or more responsive (at most 0.05 smaller effect size) than the SF-PF subscale of the SF-36 between the consecutive time points		
**T2-T3**	**PROMIS-PF: 0.51** **5.08 (10.01)**	**SF-PF: 0.53** **11.21 (21.26)**
Total hypotheses confirmed: 6/7 = 85.7%		
B. PROMIS-APS		
Hypotheses on each time point	Explanation Group or subscale: Effect size Change score (SD)	
Hypothesis: Intermediate surgical procedures show larger change in participation scores between the consecutive time points than minor surgical procedures		
**T0-T1**	**Intermediate: 1.20** **11.81 (9.82)**	**Minor: 0.68** **6.97 (10.12)**
T1-T2	Intermediate: 0.25 2.56 (10.04)	Minor: 0.42 5.06 (12.04)
**T2-T3**	**Intermediate: 1.16** **8.78 (7.54)**	**Minor: 0.45** **5.55 (12.36)**
Hypothesis: The PROMIS-APS is equally or more responsive (at most 0.05 smaller effect size) than the WHO-LA-H of the WHODAS between the consecutive time points		
T0-T1	PROMIS-APS: 0.90 9.07 (10.06)	Who-LA-H: 2.10 51.67 (24.37)
T1-T2	PROMIS-APS: 0.33 3.98 (11.95)	Who-LA-H: 1.17 34.00 (29.13)
**T2-T3**	**PROMIS-APS: 0.59** **7.00 (11.91)**	**Who- LA-H: 0.32** **10.00 (31.58)**
Hypothesis: The PROMIS-APS is equally or more responsive (at most 0.05 smaller effect size) than the WHO-LA-W of the WHODAS between the consecutive time points		
T0-T1	PROMIS-APS: 0.90 9.07 (10.06)	WHO-LA-W: 1.79 51.42 (28.80)
T1-T2	PROMIS-APS: 0.33 3.98 (11.95)	WHO-LA-W: 0.96 30.24 (31.47)
**T2-T3**	**PROMIS-APS: 0.58** **7.00 (11.91)**	**WHO-LA-W: 0.30** **11.08 (36.47)**
Hypothesis: The PROMIS-APS is equally or more responsive (at most 0.05 smaller effect size) than the WHO-PART of the WHODAS between the consecutive time points		
**T0-T1**	**PROMIS-APS: 0.90** **9.07 (10.06)**	**WHO-PART: 0.74** **16.11 (21.70)**
T1-T2	PROMIS-APS: 0.33 3.98 (11.95)	WHO-PART: 0.70 13.06 (18.61)
**T2-T3**	**PROMIS-APS: 0.58** **7.00 (11.91)**	**WHO-PART: 0.49** **9.78 (20.00)**
The PROMIS-APS is equally or more responsive (at most 0.05 smaller effect size) than the SF-PRF subscale of the SF-36 between the consecutive time points (T2-T3)		
**T2-T3**	**PROMIS-APS: 0.58** **7.00 (11.91)**	**SF-PRF: 0. 23** **9.48 (40.97)**
Total hypotheses confirmed: 7/13 = 53.8%		

Correct predicted hypotheses are highlighted in bold

## Discussion

### Main findings

In this pilot study we evaluated the construct validity and responsiveness of two different PROMIS short forms. The construct validity and responsiveness of the PROMIS-PF were supported by the data. The construct validity as well as the responsiveness of the PROMIS-APS were not supported by the data.

### Interpretation

PROMIS is increasingly being used in clinical populations, including patients undergoing surgical procedures. Especially in orthopedic surgery PROMIS instruments are widely applied. They are used pre-operatively as a predictor for postoperative improvement, or postoperatively to measure outcomes [32–39]. As far as we are aware, PROMIS was used in only one study in patient undergoing abdominal surgery [40]. In this study several PROMIS short forms were evaluated in several patient groups, including patients who underwent hernia inguinal surgery. The results of this study supported the ability of PROMIS instruments to detect week to week changes, however no other validated instruments were used so construct validity could not be evaluated. In orthopedic surgery, several studies have been performed evaluating PROMIS [38, 39, 41]. Owen Papuga et al. evaluated the English PROMIS PF CAT to asses physical function outcomes after anterior cruciate ligament (ACL) reconstructive surgery [38]. Remarkable in this study was that improvement in physical function was detected until 52 weeks after surgery with the PROMIS-PF, but not with the other measurement instruments which were used in this study (GAITRite walk testing, and IKDC assessment of knee function). This is in line with what we found in our study: between the final measurement moments, we found higher effect sizes for both PROMIS questionnaires than for the subscales of the WHODAS and SF-36. A likely explanation is that PROMIS measures are more precise at the extremes of the scale [42], which means that they can better measure higher levels of function and participation.

### Strengths and limitations

Strengths of this study are the high response rate and small number of missing values. In addition, we used hypothesis based testing, which is the state of the art methodology for evaluating the construct validity and responsiveness of a questionnaire. However this study also has some limitations. First of all, the sample size was rather small (*n* = 30), which means that the observed correlations may not be very reliable. This could be an explanation for the variation in observed correlations at different time points. The sample size was rather small because the study was not designed as a validation study, but as a feasibility study for using an accelerometer [19]. However, we considered the data interesting enough for validity and responsiveness analyses because PROMIS was not validated in this population before and responsiveness was not yet evaluated in Dutch patients at all. Second, the expected correlations were opinion-based, rather than evidence-based. It might be argued that our hypotheses were too strict. We expected high correlations (> 0.7) between the measurement instruments because the instruments aim to measure the same construct. However, there are still slight differences in the actual constructs being measured. For example, the PROMIS-APS measures the ability to participate in social roles and activities by asking e.g. “I have to limit social activities outside my home” while the WHODAS measures the perceived difficulty in participation by asking e.g. “how much of a problem did you have in joining in community activities”. Maybe these differences in construct are larger than we expected. We also did not take measurement error into account in our hypotheses, while measurement error causes weakening of correlations between scores. This could especially have played a role in the correlations between change scores, because measurement error is included twice, so the observed correlations may be smaller than expected based on the constructs being measured. Finally, it cannot be ruled out that the PROMIS-APS short form is not as responsive as expected. Even though the correlations of the PROMIS-APS with the other measurement instruments were not as high as expected, they pointed in the right direction and were still moderately high (> 0.55). Taking the differences in constructs into account, we think the results are reassuring enough to consider further testing of the CAT version, to evaluate if the CAT version is more responsive.

### Implications for clinical practice and research

PROMIS has a number of advantages over traditional questionnaires, one of them being that it offers a system of instruments, measuring different aspects of health (not only physical function and participation, but also e.g. pain interference, fatigue, sleep disturbances, anxiety and depression). These are commonly measured constructs, which can be measured with PROMIS instruments in a standardized way across disease populations. Currently, many different instruments are used for measuring postoperative recovery. The use of PROMIS will enhance the interpretability and comparability of study results. Another advantage is that PROMIS scores are expressed as T-scores, relative to the general population, which may be valuable for interpreting postoperative scores for curative procedures. The most important advantage of PROMIS instruments however, is that they can be administered as CAT [43]. The main advantage is that fewer questions are required to get a reliable score. On average 5–7 items are required with CAT to get a score with equal reliability to a 20–30 item questionnaire. Furthermore, patients will get more relevant questions because their answers to previous questions are taken into account. This makes PROMIS highly suitable to assess different components of the recovery process in the least time consuming way. CAT software for using PROMIS in the Netherlands was not yet available at the start of our study, but it it is now. We therefore recommend studies with larger sample sizes using different PROMIS item banks and CATs to evaluate the applicability of PROMIS in postoperative care. In addition, we recommend to also include patients undergoing open abdominal surgical procedures in future studies. These procedures were not included in the current study, since this study was conducted as a pilot in study in preparation for a clinical trial in which only laparoscopic procedures would be included [19, 20].

## Conclusions

This study supported the construct validity and the responsiveness of the PROMIS-PF v1.2 short form 8b for measuring recovery in abdominal surgery. Considering the major advantages of PROMIS, we recommend the use of the PROMIS-PF in abdominal surgery. Even though the correlations of the PROMIS-APS v2.0 short form 8a with the other measurement instruments were not as high as expected, they were still moderately high (> 0.55) and further testing is recommended.

## Abbreviations

CAT: Computerized Adaptive Testing
IRT: Item Response Theory
PROMIS-APS: PROMIS Ability to Participate in Social Roles and Activities v2.0 short form 8a
PROMIS-PF: PROMIS Physical Function v1.2 short form 8b
SD: Standard deviation
SF-36: Short Form 36 Health Survey
SF-BP: Bodily Pain
SF-ERF: Emotional Role Functioning
SF-GH: General Health
SF-MH: Mental Health
SF-PF: Physical Functioning
SF-PRF: Physical Role Functioning
SF-SF: Social Functioning
SF-VT: Vitality
WHO-CG: Cognition – understanding & communicating
WHODAS: World Health Organization Disability Assessment Schedule 2.0
WHO-GA: Getting Along– interacting with other people
WHO-LA: Life Activities– domestic responsibilities, leisure, work & school
WHO-LA-H: Life Activities subscale - Household
WHO-LA-W: Life Activities subscale - Work
WHO-MO: Mobility– moving & getting around
WHO-PART: Participation– joining in community activities
WHO-SC: Self-Care– hygiene, dressing, eating & staying alone
